# Sotorasib in KRAS^G12C^ mutated lung cancer: Can we rule out cracking KRAS led to worse overall survival?

**DOI:** 10.1016/j.tranon.2022.101591

**Published:** 2022-12-26

**Authors:** Timothée Olivier, Alyson Haslam, Vinay Prasad

**Affiliations:** a Department of Oncology, Geneva University Hospital, 4 Gabrielle-Perret-Gentil Street, 1205, Geneva, Switzerland; b Department of Epidemiology and Biostatistics, University of California San Francisco, 550 16th St, 2nd Fl, San Francisco, CA 94158, USA

**Keywords:** Non-small cell lung cancer, KRAS inhibitors, Biomarker, Informative censoring, Power analysis

## Abstract

The *KRAS* oncogene is present in up to 25% of solid tumors and for decades had been undruggable. Sotorasib was the first-in-class KRAS inhibitor to reach the US and European market, and its pharmacological inhibition is restricted to the KRAS p.G12C mutation. Sotorasib showed activity (tumor shrinkage) in patients with non-small cell lung cancer harboring this specific mutation, and efficacy was tested in the CodeBreaK 200, open-label, phase 3 trial (NCT04303780). The results were presented in the ESMO 2022 meeting. CodeBreaK 200 found an improvement in the primary endpoint of progression-free survival (PFS), but overall survival, a key secondary endpoint, was not improved. However, critical questions about the trial's design may limit inferences regarding the reported results. The control arm treatment was inferior to the best standard of care. A late protocol modification (which lowered the sample size and allowed a problematic crossover) prohibited the trial from making a determination regarding overall survival. Imbalance in censoring rates, with potential informative censoring, makes PFS estimates unreliable. Quality-of-life data were also limited. Ultimately, CodeBreaK 200 does not clarify how this therapy should be used in practice, and while we maintain cautious enthusiasm for this and other Ras inhibitors, we await more informative trials

## Highlights

• Sotorarib was the first KRAS G12C inhibitor to be approved in NSCLC.
• CodeBreaK 200 was a phase 3 trial of sotorasib versus docetaxel in the second-line setting.
• The control arm was beneath the best standard-of-care, cross-over was problematic.
• High rates of censoring make progression-free survival estimates unreliable.
• The sample size was lowered: an overall survival decrement cannot be excluded.

## Graphical abstract

**Figure:**
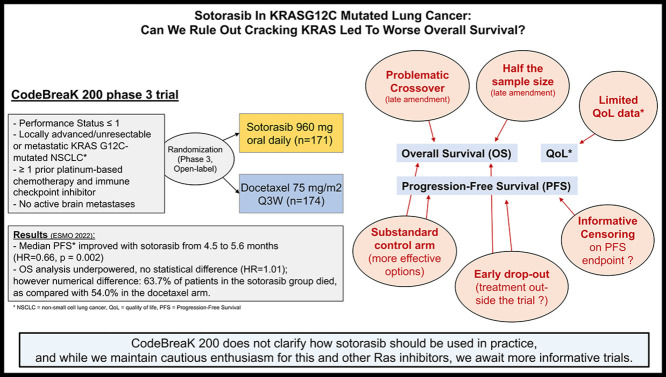

## Introduction

The *KRAS* oncogene (*Kirsten rat sarcoma viral oncogene*) encodes for the mutated KRAS oncoprotein which is present in up to 25% of solid tumors, and for decades had been undruggable. [1] Sotorasib was the first-in-class KRAS inhibitor to reach the US and European market, which locks the KRAS mutated protein in its inactive state. Sotorasib pharmacological inhibition is restricted to the KRAS p.G12C mutation, which is found at a frequency of 14% in non-small cell lung cancer (NSCLC) tumors and varying frequencies in other tumors [2]. However, the prevalence varies according to ethnicity and other population characteristics, as seen with a lower 8% rate in Colombia, with important variations across regions [3].

Based on the results of the single-arm, multicenter, phase I/II CodeBreaK 100 study (NCT03600883), sotorasib received accelerated approval from the US FDA in May 2021 for “adult patients with KRAS G12C‑mutated locally advanced or metastatic non-small cell lung cancer (NSCLC), as determined by an FDA‑approved test, who have received at least one prior systemic therapy”. An overall response rate of 41% with a 12.3 months median duration of response was seen in the most recent update analysis [4]. Sotorasib clearly has drug activity—capable of shrinking Ras dependent tumors—yet at the time of approval, efficacy, e.g., in which settings and circumstances it could be clinically meaningful for patients with KRAS^G12C^ NSCLC, had not been shown.

CodeBreaK 200 (NCT04303780) set out to address efficacy. It is a phase 3, open-label, global trial, randomizing patients with locally advanced or metastatic KRAS^G12C^ mutated NSCLC to sotorasib or docetaxel [5]. Patients must have previously received a platinum-doublet chemotherapy and checkpoint inhibitor.

CodeBreaK 200 found an improvement in the primary endpoint of progression-free survival (PFS), but overall survival (OS), a secondary endpoint, was not improved [6]. While we welcome these results, critical questions about the trial's design remain. Specifically, questions of power, crossover, informative censoring and quality-of-life (QoL) limit inferences regarding the trial.

## Control arm and eligibility

When it comes to subsequent therapies in NSCLC, either we accept PFS as the benchmark to change practice, or we accept OS. Proponents of CodeBreaK 200 argue PFS is sufficient to change standard-of-care, but by this logic, the control arm is inadequate.

At least two other regimens have had improved PFS over docetaxel in phase 3 trials. In the IFCT-1103 ULTIMATE study, patients with non-squamous NSCLC (the histology mostly affected by KRAS mutations) after one to two previous lines of therapy, including one line of platinum-doublet chemotherapy, were randomized between paclitaxel plus bevacizumab or docetaxel [7]. A PFS benefit in the combination treatment was demonstrated, with a median improvement from 3.9 to 5.4 months. The hazard ratio (HR=0.61, 95% [CI]: 0.44, 0.86; *p* = 0.005) indicated that the benefit was at least as high, if not higher, as in CodeBreaK 200 (HR for PFS=0.66, 95% [CI]: 0.51, 0.86; *p* = 0.002).

In the REVEL trial, docetaxel plus ramucirumab has also shown superior PFS as compared with docetaxel in the second-line of patients with NSCLC progressing during or after a platinum-based chemotherapy. The median PFS improvement was 3.0 to 4.5 months (HR=0.76, 95% [CI]: 0.68, 0.86; *p*<0.0001) [8]. More importantly, the REVEL trial has also demonstrated survival gain. As of 2014, REVEL found OS benefit from 9.1 months to 10.5 months (HR=0.86, 95% [CI]: 0.75, 0.98; *p* = 0.023). These results were known 72 months before the enrollment on CodeBreaK 200, and as such, the CodeBreaK 200 trial should have used ramucirumab plus docetaxel (or paclitaxel plus bevacizumab) as the control arm.

## Protocol amendment

CodeBreaK 200 accrued patients between June 2020 and April 2021. A protocol amendment occurred on February 2021 with 2 implications for the trial. First, the sample size was reduced. The anticipated number of patients (as defined in the protocol) was changed from 650 in the initial design to 330 patients [9]. This amendment occurred “per regulatory guidance” [6]. However, the authors did not explain the basis for this. Notably, this change likely preserved the study power to detect a PFS benefit—under most reasonable assumptions—but lost study power to evaluate OS, a secondary endpoint. Next, the trial was modified to permit crossover, which allowed a significant proportion of patients to receive sotorasib upon progression. Both actions ensured that CodeBreaK 200 could not establish or exclude a survival gain or even decrement.

## The case against crossover

In CodeBreaK 200, crossover was allowed *per protocol* after this amendment, and 26.4% of control arm patients ultimately received sotorasib, resulting in 34% of patients in the control arm receiving a subsequent KRAS inhibitor.

Crossover can be desirable or problematic in different situations in oncology [10]. CodeBreaK 200 represents a problematic use because the trial tests – for the first time – the fundamental efficacy of the drug. Prior to this study, no trial has shown sotorasib improves clinical outcomes in randomized fashion.

When crossover occurs and no survival benefit is seen (like in CodeBreaK 200), many argue that OS results were “confounded” by crossover. In other words, because patients in the control arm later received the experimental drug, both arms benefited from receiving the new therapy at some point, and as a result, any difference in OS could not be captured. But this reasoning is flawed. It assumes that sotorasib is a life-extending therapy and fails to consider alternative explanations.

There are 3 equally valid interpretations when OS has not been shown in presence of problematic crossover – 1) the drug has clinical efficacy, but the gain was masked; 2) the drug is detrimental, and the harm was masked; and 3) the drug has no effect on clinical efficacy.

If the experimental drug has efficacy, and crossover was sufficient to salvage OS, one could argue the new therapy may be given upfront or later during the course of treatment (the crossover). Alternatively, sotorasib could be a drug that shortens OS. By allowing crossover, the detrimental effect in the experimental arm will also affect the control arm, and potential differences may not be observed. In this setting, similar OS may hide a detrimental effect.

Because crossover occurred in CodeBreaK 200, and the efficacy of sotorasib was not shown, it is impossible to know which of these 3 scenarios occurred. Some may argue that it would have been unethical to deny sotorasib to a patient after progression in the control arm of the CodeBreaK 200 trial. However, a pre-requisite ethical principle when running a trial is that the trial should ultimately answer meaningful question [11]. If the design fails to do so, then it is itself unethical.

By changing the sample size and permitting crossover, investigators essentially set up CodeBreaK 200 to be a trial that could not find a survival gain or exclude a survival decrement, if it exists.

## Original power and new power

Dramatic change in sample size has several implications for results. First, the trial initial statistical plan was “not powered to detect a statistical difference” in OS, but the reduction in sample size (from 650 to 330) ensured lack of power to detect a survival benefit or even decrement [12].

If sotorasib is a detrimental drug (which may or may not be), the unexplained reduction in sample size could have ensured that a worse survival with sotorasib would not be detected. Indeed, a numerical signal of worse survival exists in the CodeBreaK 200 trial: 63.7% of patients in the sotorasib group died, as compared with 54.0% in the docetaxel arm, yet the HR was 1.01, and the confidence interval was wide.

Some argue, based on better safety profile and QoL over docetaxel, that sotorasib may be justifiable even without an OS benefit. However, if we were to establish sotorasib is *as good as* docetaxel, but better tolerated, this question would be best answered by a non-inferiority trial design. Based on the reported OS result of the CodeBreaK 200 trial, and using a loose non-inferiority margin of 1.3 (one-sided alpha=0.025, power=80%, 36 months accrual and 48 months follow-up), we calculated that the sample size to demonstrate non-inferiority would be 2076 patients [13,14].

## Limitation in quality-of-life data

In CodeBreaK 200, the change in QoL was analyzed from baseline to week 12, a limited period which cannot capture the entire patient experience. A patient treated with sotorasib may receive docetaxel as a salvage therapy upon progression, and QoL during this phase of therapy will be missing in the CodeBreaK 200 analysis. Analysis of a limited period of time surely lacks information on what matters to patient: their QoL over all lines of treatment and beyond [15]. Second, financial toxicity is not or poorly captured in RCTs, for the simple reason that the cost of drugs is often supplied by the sponsor. The cost of 21 days of sotorasib is currently $ US 15 952 (according to the Redbook), when one cycle of docetaxel (every 21 day) may cost as low as $ US 161 (for a 140 mg dose), which is 99 times cheaper.

## Informative censoring may have occurred in the PFS analysis

The CodeBreaK 200 trial found a median improvement in PFS from 4.5 to 5.6 months (less than 5 weeks; HR=0.66, *p* = 0.002). Yet, at least part of this improvement could be due to informative censoring.

After reconstructing individual patient data from the presented Kaplan Meier curves of the CodeBreaK 200 trial, following a methodology previously described [16], we estimated 16% of patients in the sotorasib arm were censored during the first 6 months, as compared with 33% of patients in the docetaxel arm. In other words, during the first months while in the trial, a higher proportion of patients in the control arm choose to stop the trial or ended follow-up before undergoing their CT scan to assess progression.

When censoring rate imbalance occurs early in a study, different explanations may be at play. Censoring may occur due to drug toxicity, with preferential censoring of the frailest individuals. If censoring is more prevalent in one arm because of an excess in toxicity, a bias is introduced favoring this arm (because events are averaged among the healthier participants who are retained).

However, another source of informative censoring is driven by patient or physician disappointment. A recent example was the VISION trial, which tested the addition of Lu-PSMA to “standard care” in patients with metastatic castrate-resistant prostate cancer [17]. Patients in the control arm received suboptimal therapy, and 56% of patients initially dropped-out [18]. This type of early drop-out has been described across tumor types, with early censoring similar to the CodeBreaK 200 trial, occurring more often in the control arm [19]. A clue indicating this phenomenon likely occurred in CodeBreaK 200 is that after randomization, 2 patients in the experimental arm did not receive sotorasib (1.2%) when 23 patients in the control arm did not receive docetaxel (13.2%) [6].

In other words, more patients enrolled in the docetaxel arm preferred to quit immediately or stop the trial early, probably to seek treatment outside the study (including sotorasib or another KRAS inhibitor). It is possible those patients were in better health condition, were more connected, and/or wealthier than those remaining in the study. This type of censoring may have resulted in frailer patients remaining in the control arm, which would artificially favor the experimental drug. Imbalances in censoring can undermine randomization itself.

## Conclusion

Sotorasib has shown promising activity, has demonstrated a PFS benefit, and represents a success of industrial chemistry—the ability to drug an aberrant gene product hitherto undruggable. But CodeBreaK 200 suffers from key limitations: a control arm beneath the best standard of care; a protocol modification (reducing sample size and allowing crossover) that prohibited the trial from making a determination regarding OS; and imbalance in censoring rates that make PFS estimates unreliable. Ultimately, CodeBreaK 200 does not clarify how this therapy should be used in practice, and while we maintain cautious enthusiasm for this and other Ras inhibitors, we await more informative trials.

## Authors contribution statement

VP and TO contributed to the conception. TO and AH contributed to the statistical analyses. TO wrote first draft of manuscript and all authors reviewed and revised the manuscript. All authors provided final approval of the manuscript.

## Funding

This project was funded by 10.13039/100014848Arnold Ventures, LLC through a grant paid to the University of California, San Francisco. The funders had no role in the design and conduct of the study.

## Declaration of Competing Interest

Vinay Prasad's Disclosures: Research funding: Arnold Ventures; Royalties: Johns Hopkins Press, Medscape; Honoraria: Grand Rounds/lectures from universities, medical centers, non-profits, and professional societies; Consulting: UnitedHealthcare; Speaking fees: Evicore; Other: Plenary Session podcast has Patreon backers. All other authors have no financial nor non-financial conflicts of interest to report.

## References

[1] Mullard A. (2019). Cracking KRAS. Nat. Rev. Drug Discov..

[2] Nassar A.H., Adib E., Kwiatkowski D.J. (2021). Distribution of KRASG12C Somatic Mutations across Race, Sex, and Cancer Type. N. Engl. J. Med..

[3] Ruiz-Patiño A., Rodríguez J., Cardona A.F. (2022). G12C KRAS mutation prevalence in non-small cell lung cancer: contribution from interregional variability and population substructures among Hispanics. Transl. Oncol..

[4] (2022). https://www.aacr.org/about-the-aacr/newsroom/news-releases/kras-g12c-inhibitor-sotorasib-may-offer-long-term-clinical-benefit-in-patients-with-non-small-cell-lung-cancer/.

[5] Reck M., Spira A., Besse B. (2021). MO01.32 CodeBreaK 200: a Phase 3 Multicenter Study of Sotorasib, a KRAS(G12C) Inhibitor, versus Docetaxel in Patients with Previously Treated Advanced Non-Small Cell Lung Cancer (NSCLC) Harboring KRAS p.G12C Mutation. J. Thorac. Oncol..

[6] Johnson M. (2022). https://oncologypro.esmo.org/meeting-resources/esmo-congress/sotorasib-versus-docetaxel-for-previously-treated-non-small-cell-lung-cancer-with-kras-g12c-mutation-codebreak-200-phase-iii-study.

[7] Cortot A.B., Audigier-Valette C., Molinier O. (2020). Weekly paclitaxel plus bevacizumab versus docetaxel as second- or third-line treatment in advanced non-squamous non-small-cell lung cancer: results of the IFCT-1103 ULTIMATE study. Eur. J. Cancer Oxf. Engl..

[8] Garon E.B., Ciuleanu T.E., Arrieta O. (2014). Ramucirumab plus docetaxel versus placebo plus docetaxel for second-line treatment of stage IV non-small-cell lung cancer after disease progression on platinum-based therapy (REVEL): a multicentre, double-blind, randomised phase 3 trial. Lancet.

[9] History of Changes for Study: NCT04303780. Accessed September 19, 2022. https://clinicaltrials.gov/ct2/history/NCT04303780?A=1&B=22&C=Side-by-Side#StudyPageTop.

[10] Haslam A., Prasad V. (2018). When is crossover desirable in cancer drug trials and when is it problematic?. Ann. Oncol..

[11] Prasad V., Grady C (2014). The misguided ethics of crossover trials. Contemp. Clin. Trials.

[12] Altman D.G., Bland J.M. (1995). Statistics notes: absence of evidence is not evidence of absence. BMJ.

[13] Nagashima K. A sample size determination tool for the log-rank test of non-inferiority [Internet]. Published May 27, 2015. https://nshi.jp/en/js/twosurvyrni/.

[14] Jung S.H., Chow S.C. (2012). On sample size calculation for comparing survival curves under general hypothesis testing. J. Biopharm. Stat..

[15] Haslam A., Herrera-Perez D., Gill J., Prasad V (2020). Patient experience captured by quality-of-life measurement in oncology clinical trials. JAMA Netw. Open.

[16] Liu N., Zhou Y., Lee J.J. (2021). IPDfromKM: reconstruct individual patient data from published Kaplan-Meier survival curves. BMC Med. Res. Methodol..

[17] Sartor O., de Bono J., Chi K.N. (2021). Lutetium-177–PSMA-617 for metastatic castration-resistant prostate cancer. N. Engl. J. Med..

[18] Olivier T., Powell K., Prasad V. (2022). Lutetium-177-PSMA-617 in metastatic castration-resistant prostate cancer: limitations of the VISION Trial. Eur. Urol..

[19] Rosen K., Prasad V., Chen E.Y. (2020). Censored patients in Kaplan–Meier plots of cancer drugs: an empirical analysis of data sharing. Eur. J. Cancer.

